# Synthesis, In Silico and In Vitro Evaluation of Antimicrobial and Toxicity Features of New 4-[(4-Chlorophenyl)sulfonyl]benzoic Acid Derivatives

**DOI:** 10.3390/molecules26165107

**Published:** 2021-08-23

**Authors:** Theodora-Venera Apostol, Mariana Carmen Chifiriuc, Constantin Draghici, Laura-Ileana Socea, Luminita Gabriela Marutescu, Octavian Tudorel Olaru, George Mihai Nitulescu, Elena Mihaela Pahontu, Gabriel Saramet, Stefania-Felicia Barbuceanu

**Affiliations:** 1 Faculty of Pharmacy, “Carol Davila” University of Medicine and Pharmacy, 6 Traian Vuia Street, 020956 Bucharest, Romania; theodora.apostol@umfcd.ro (T.-V.A.); laura.socea@umfcd.ro (L.-I.S.); george.nitulescu@umfcd.ro (G.M.N.); elena.pahontu@umfcd.ro (E.M.P.); gabriel.saramet@umfcd.ro (G.S.); stefania.barbuceanu@umfcd.ro (S.-F.B.); 2 Department of Botany and Microbiology, Faculty of Biology, University of Bucharest, 1-3 Aleea Portocalelor, 60101 Bucharest, Romania; carmen.chifiriuc@bio.unibuc.ro; 3 “Costin D. Nenițescu” Centre of Organic Chemistry, Romanian Academy, 202 B Splaiul Independenței, 060023 Bucharest, Romania; cst_drag@yahoo.com

**Keywords:** *N*-acyl-α-amino acid, 4-isopropyl-1,3-oxazol-5(4*H*)-one, *N*-acyl-α-amino ketone, 4-isopropyl-1,3-oxazole, antimicrobial activity, antibiofilm agents, toxicity

## Abstract

The multi-step synthesis, physico-chemical characterization, and biological activity of novel valine-derived compounds, i.e., *N*-acyl-α-amino acids, 1,3-oxazol-5(4*H*)-ones, *N*-acyl-α-amino ketones, and 1,3-oxazoles derivatives, bearing a 4-[(4-chlorophenyl)sulfonyl]phenyl moiety are reported here. The structures of the newly synthesized compounds were confirmed by spectral (UV-Vis, FT-IR, MS, ^1^H- and ^13^C-NMR) data and elemental analysis results, and their purity was determined by RP-HPLC. The new compounds were assessed for their antimicrobial activity and toxicity to aquatic crustacean *Daphnia magna*. Also, in silico studies regarding their potential mechanism of action and toxicity were performed. The antimicrobial evaluation revealed that the 2-{4-[(4-chlorophenyl)sulfonyl]benzamido}-3-methylbutanoic acid and the corresponding 1,3-oxazol-5(4*H*)-one exhibited antimicrobial activity against Gram-positive bacterial strains and the new 1,3-oxazole containing a phenyl group at 5-position against the *C. albicans* strain.

## 1. Introduction

The rapid rise of infectious diseases has become one of the biggest potential threats to human health, particularly because of the emergence of resistance to current antimicrobial drugs. In this context, there is a worldwide interest for the discovery of new antimicrobial agents with increased effectiveness, especially on microbial strains resistant to all current antibiotics.

Microorganisms (bacteria, fungi, protozoa) and viruses comprise the majority of terrestrial biodiversity, are an integral part of biosphere processes, and often adhere to different surfaces, forming biofilms. Thus, for example, bacteria possess specialized surface structures generically called adhesins, capable of interacting stereospecifically with receptors on the host cell membrane, similar to antigen-antibody or lectin-carbohydrate interaction. Adhesion to a substrate is the initial and essential stage in the formation of a microbial biofilm, which shows increased resistance to both host defense mechanisms and conventional doses of antibiotics and biocides, the cells exhibiting the property of tolerance or phenotypic resistance to antimicrobial substances. Available antibiofilm strategies involve the use of agents that either inhibit or kill microorganisms or target the expression of adhesins and of genes associated with biofilm development and/or of their regulators [1,2].

**Figure 1:**
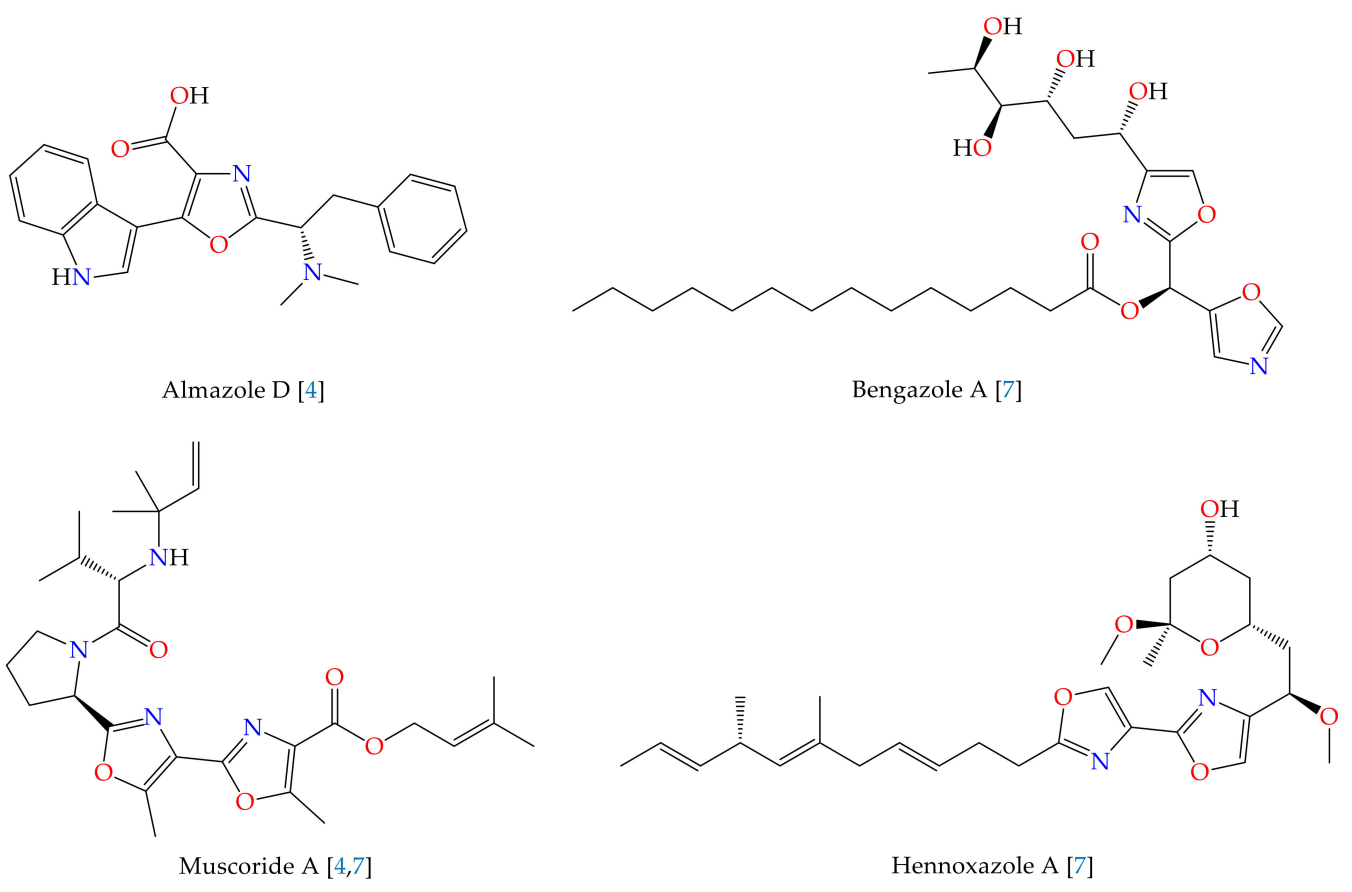
Structures of some representative bioactive naturally occurring compounds containing 1,3-oxazole moiety.

In this study, we started from the premise that the 1,3-oxazole nucleus is an important scaffold in synthetic, pharmaceutical, and medicinal chemistry, with a remarkable versatility that results in a high structural diversity. Also, it is well known that numerous naturally occurring heterocycles bearing 1,3-oxazole cores are bioactive substances, exhibiting many biological properties [3,4,5,6,7], of which it is worth mentioning their antibacterial (e.g., almazole D, muscoride A), antifungal (e.g., bengazole A, mycalolide A), antiviral (e.g., hennoxazole A), and antiproliferative (e.g., bistratamide M and N, diazonamides A–E) properties. The structures of representative naturally occurring bioactive compounds sharing the 1,3-oxazole template are presented in Figure 1.

**Figure 2:**
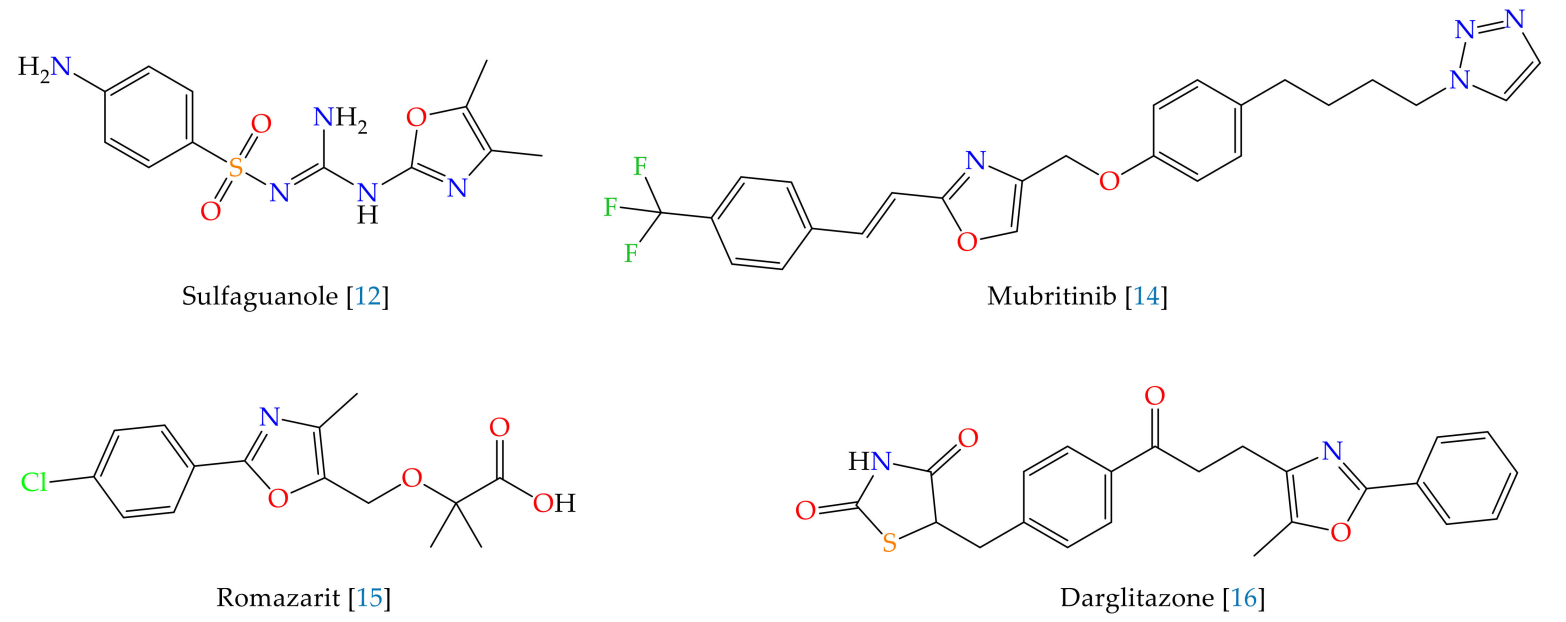
Structures of some representative bioactive synthesized compounds containing 1,3-oxazole moiety.

The literature survey on the synthesized five-membered heterocycles with two heteroatoms from 1,3-oxazoles class highlighted that they are also endowed with a wide range of potent pharmacological activities [8,9,10,11,12,13,14,15,16], such as antifungal, antibacterial (e.g., sulfaguanole with trade name: Enterocura, a sulfanilamide drug used for patients with acute diarrhea), anticancer (e.g., mubritinib), analgesic, antipyretic, anti-inflammatory (e.g., oxaprozin, a COX-2 inhibitor currently in the market, romazarit, the Roche candidate, which was withdrawn due to its toxicity profile), and anti-diabetic (e.g., darglitazone, an antihyperglycemic agent belonging to the glitazone class) effects. The structures of the representative bioactive synthetic compounds sharing the 1,3-oxazole scaffold are presented in Figure 2.

Whereas the 1,3-oxazole moiety is a common heterocyclic motif identified in numerous natural products and biologically active synthetic molecules, over time the preparation of compounds from this class has attracted the attention of researchers who have reported many efficient chemical methods of synthesis. The most important synthetic strategies of 1,3-oxazoles include the reaction of aldehydes with tosylmethyl isocyanide (van Leusen synthesis), the Cornforth rearrangement, the condensation of aldehydes with cyanohydrins in the presence of hydrogen chloride (Fischer synthesis), the Doyle reaction of diazocarbonyl compounds with nitriles, the Robinson–Gabriel synthesis consisting of the intramolecular cyclodehydration of *N*-acyl-α-amino ketones, the Blümlein–Lewy method involving the condensation of α-halo or α-hydroxy ketones with primary amides, and the Dakin–West reaction of α-amino acids with acid anhydrides in the presence of a base, typically pyridine [9,17,18], etc. Recently, synthesis of the 2,5-polyfunctionalized 1,3-oxazole derivatives starting from acyl chlorides and propargyl amine via a consecutive amidation-coupling-isomerization sequence involving an in situ acyl Sonogashira cross-coupling was developed by Müller’s group [19]. The 2,4- and 2,4,5-substituted 1,3-oxazoles were obtained from a wide range of α-bromo ketones and amides via a silver triflate mediated cyclization [20] and the sonochemical synthesis of the polyarylated 1,3-oxazoles was achieved via the reaction of benzylamines with benzoin in the presence of 2-iodoxybenzoic acid [21].

The 1,3-oxazol-5-ols exist in their corresponding 5-oxo tautomer, namely 1,3-oxazol-5(4*H*)-ones, which are also reported to display antimicrobial (e.g., jadomycin B), antiviral,and trypanocidal properties [22,23,24]. In addition, the saturated azlactones are widely used as intermediates in the synthesis of different heterocyclic scaffolds (including, 1,3-oxazoles, pyrroles, pyrrolines, imidazoles, and imidazolines) or of acyclic derivatives with potential biological properties [25,26]. Thus, due to their synthetic and biological importance, several methods of preparing 1,3-oxazol-5(4*H*)-ones have been developed, the most widely used being the intramolecular cyclization of *N*-acyl-α-amino acids mediated by a carboxylic group activator, including acetic anhydride, *N*,*N*′-dicyclohexylcarbodiimide, 1-ethyl-3-(3-dimethylaminopropyl)carbodiimide, chloromethylenedimethylammonium chloride (Vilsmeier reagent), and alkyl chloroformates in the presence of *N*-methylmorpholine [27,28,29,30,31,32,33,34], etc. Fujita and Kunishima used a one-pot synthesis of 1,3-oxazol-5(4*H*)-ones by the *N*-acylation of α-amino acids with carboxylic acids and the subsequent cyclodehydration of the resulting *N*-acyl-α-amino acids by the addition of *N*,*N*-diethylaniline. Both these reactions proceed effectively with the same reagent, 4-(4,6-dimethoxy-1,3,5-triazin-2-yl)-4-methylmorpholinium chloride, in aqueous solvents [35]. Wen et al. developed a new method to prepare 4-acetoxy substituted 5(4*H*)-oxazolones by direct oxidation of *N*-benzoyl-α-amino acids using hypervalent iodine [36]. Recently, a series of 1,3-oxazol-5(4*H*)-one derivatives were generated exclusively in the palladium-catalyzed tunable imidoylative reaction of allyl-2-benzyl(or allyl)-2-isocyanoacetates with aryl triflates as electrophiles [37].

Also, many acyclic intermediates from the class of *N*-acyl-α-amino acids are active pharmaceutical ingredients with a wide spectrum of therapeutic properties [38,39,40,41,42], like anticancer (e.g., ubenimex, also known as bestatin), antioxidant, mucolytic (e.g., *N*-acetyl cysteine), antihypertensive (e.g., enalapril, an angiotensin-converting enzyme inhibitor), vasoconstrictor (e.g., angiotensin II), anti-ulcer (e.g., benzotript), and are also used for the treatment of acetaminophen overdose (e.g., *N*-acetyl cysteine). Further, the synthesis of various analogs of *N*-acyl-α-amino acids derived from fatty acids (also known as elmiric acids or lipoamino acids) has been achieved and their physiological functions as endogenous signaling molecules and their pharmacological properties were studied to define receptors and other proteins that bind to them and to discover their applicability to the treatment of various diseases. For example, it was discovered that *N*-arachidonoylglycine and *N*-linoleoylalanine are anti-inflammatory, *N*-stearoylproline exhibits antimicrobial effect, *N*-oleoylserine is capable of regulating osteoblast activity, *N*-arachidonoylalanine and *N*-palmitoyltyrosine have antiproliferative action. Furthermore, *N*-acyltyrosines were identified as bacterial metabolites which display antibiotic activity against *Bacillus subtilis* and moderately inhibit the *Pseudomonas aeruginosa* biofilm formation [43], and *N*-arachidonoylserine exhibits antimicrobial and antibiofilm effects against methicillin-resistant *Staphylococcus aureus* strains [44]. The general method for obtaining *N*-acyl α-amino acids is the non-selective Schotten–Baumann type reaction of the natural or unnatural α-amino acids with acyl chlorides under basic conditions by using sodium hydroxide, sodium carbonate, or potassium carbonate. Recently, Bourkaiba et al. provided an alternative selective enzymatic synthesis by using the aminoacylases from *Streptomyces ambofaciens* to catalyze the acylation reaction of the proteinogenic amino acids with middle and long-chain fatty acids in the aqueous medium [45].

Moreover, the *N*-acyl-α-amino ketones are important in medicinal chemistry being known to present antiviral (e.g., rupintrivir), antithrombotic, antihypertensive (e.g., keto-ACE), anti-inflammatory effects and are identify as ecdysone agonists for control of gene expression [46,47,48,49,50]. The *N*-protected α-amino ketones are also versatile building blocks in the synthesis of various potential biologically active compounds, including 1,3-oxazoles and 2-oxazolines [33,51]. Therefore, over time several methodologies have been reported for their synthesis, the primary method being the Friedel–Crafts aminoacylation reaction of various aromatic compounds with *N*-acyl-α-amino acids, *N*-protected α-amino acyl chlorides, α-amino acid anhydrides, or 1,3-oxazol-5(4*H*)-ones in the presence of Lewis acids or Brönsted acids as catalysts [33,52,53,54,55,56]. Also, some functionalized derivatives were obtained by reaction of enolizable cyclic 1,3-dicarbonyls with 1,3-oxazol-5(4*H*)-ones [57]. DiRocco and Rovis reported the direct asymmetric coupling of aliphatic aldehydes and *N*-*tert*-butoxycarbonyl-protected imines in a cross-aza-benzoin reaction and obtained the corresponding *N*-protected α-amino ketones [58].  Xu et al. prepared chiral *N*-protected α-amino ketones via a highly enantioselective N-H insertion reaction of α-aryl α-diazo ketones by using cooperative catalysis by dirhodium(II) carboxylates and chiral spiro phosphoric acids [59]. Recently, Kawasaki et al. reported a photoinduced dehydrogenative coupling reaction between substituted amides and aldehydes in the presence of iridium catalyst to produce *N*-protected α-amino ketones [60], and Raclea et al. developed a silver-mediated synthesis of these compounds via the oxidative deconstruction of azetidinols using a readily scalable protocol [61].

In addition, there is increasing interest in exploring diaryl sulfones as potential pharmaceuticals, since a large number of compounds from this class are reported to have biological activities, including antimicrobial and anticancer properties [62,63]. Moreover, numerous compounds containing this pharmacophore linked by different other moieties are also exhibiting promising activities [64,65].

Given the therapeutic potential of these scaffolds, that the majority have also shown antimicrobial properties and in the continuation of our research [66,67,68,69,70,71], the purpose of this present work was to obtain and characterize new synthetic analogs from *N*-acyl-α-amino acids, 1,3-oxazol-5(4*H*)-ones, *N*-acyl-α-amino ketones, and 1,3-oxazoles classes derived from valine with a 4-[(4-chlorophenyl)sulfonyl]phenyl moiety and to evaluate their antimicrobial effect and toxic potential.

## 2. Results

### 2.1. Chemistry

#### 2.1.1. Chemical Synthesis

**Scheme 1:**
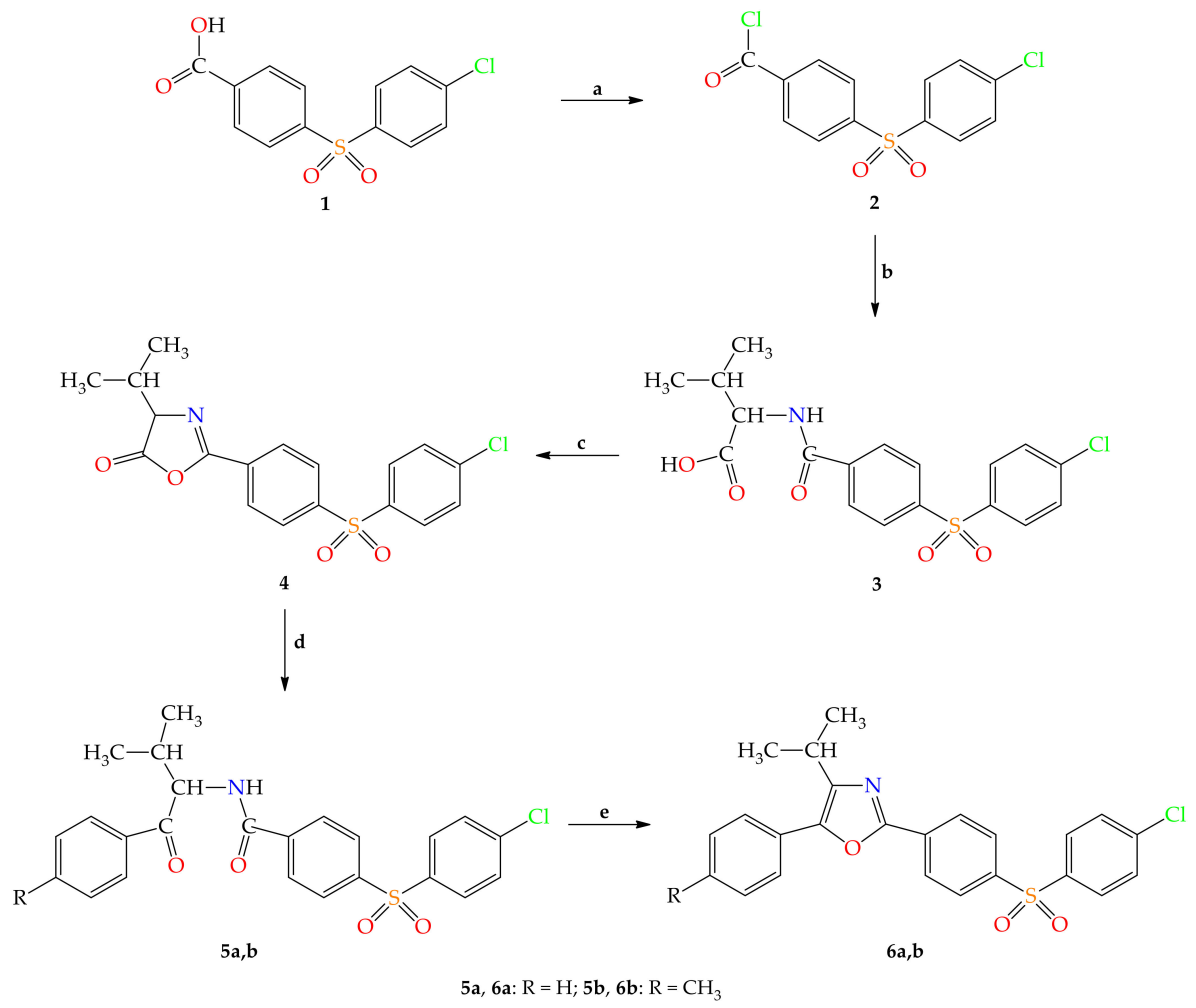
Synthesis of the new compounds 3–6. Reagents and conditions: (**a**) SOCl_2_, reflux, 30 h (99% [67]); (**b**) (**i**) valine/NaOH, CH_2_Cl_2_, 0–5 °C, 30 min; room temperature (r.t.), 1 h; (**ii**) HCl (93%); (**c**) ethyl chloroformate/4-methylmorfoline, CH_2_Cl_2_, r.t., 30 min (93%); (**d**) C_6_H_5_-R (R = H or CH_3_), AlCl_3_, r.t., 20 h (**5a**: 86%, **5b**: 90%); (**e**) POCl_3_, reflux, 4 h (**6a**: 90%, **6b**: 91%).

The new 4-[(4-chlorophenyl)sulfonyl]benzoic acid derivatives **3**–**6** were obtained using the chemical reactions presented in Scheme 1.

The synthesis of the new compounds started from the known raw material 4-[(4-chlorophenyl)sulfonyl]benzoic acid **1** which was obtained in two stages. By AlCl_3_-catalysed sulfonylation of chlorobenzene with 4-methylbenzene-1-sulfonyl chloride, at reflux, 1-chloro-4-tosylbenzene was prepared. This sulfone was oxidated by heating at reflux with CrO_3_ in glacial acetic acid to compound **1** [72]. Then, the reaction of carboxylic acid **1** with thionyl dichloride produced 4-[(4-chlorophenyl)sulfonyl]benzoyl chloride **2** [67,73], which was used crude in the *N*-acylation reaction of valine to new 2-{4-[(4-chlorophenyl)sulfonyl]benzamido}-3-methylbutanoic acid **3** in 93% yield. Subsequently, *N*-acyl-α-amino acid **3** was converted by cyclodehydration with ethyl chloroformate in presence of 4-methylmorpholine to new 2-{4-[(4-chlorophenyl)sulfonyl]phenyl}-4-isopropyl-1,3-oxazol-5(4*H*)-one **4** with a reaction yield of 93%. Then, aromatic hydrocarbons (benzene, toluene) were acylated with 1,3-oxazol-5(4*H*)-one **4** in the presence of aluminum trichloride to new *N*-(1-aryl-3-methyl-1-oxobutan-2-yl)-4-[(4-chlorophenyl)sulfonyl]benzamides **5a**,**b** in about 88% yield. Robinson–Gabriel cyclization of *N*-acyl-α-amino ketones **5a**,**b** with phosphoryl trichloride by heating at reflux gave the new 5-aryl-2-{4-[(4-chlorophenyl)sulfonyl]phenyl}-4-isopropyl-1,3-oxazoles in yield of 90 (**6a**) or 91% (**6b**). The newly synthesized compounds were characterized by spectral (NMR, FT-IR, UV-Vis, MS) and elemental analyses.

#### 2.1.2. Spectral Characterization

##### UV-Vis Spectral Data

In general, the UV-Vis absorption spectra of new compounds **3**–**6** showed two peaks at 202.6 nm (E band) and in the region of 248.5–252.9 nm (B band). 1,3-Oxazol-5(4*H*)-one **4** absorbed also at 222.9 nm (K band). Due to the presence of the 1,3-oxazole chromophore, spectra of **6a,b** showed an additional absorption band at λ_max_ = 333.9 or 337.4 nm.

##### IR Spectral Data

The IR spectra of acyclic precursors **3** and **5a,b** presented a representative absorption peak in the range of 3279–3358 cm^−1^ due to the valence vibration of the N-H bond. In the IR spectrum of **3**, the absorption band due to carbonyl stretching vibration was observed at 1737 cm^−1^, and the peak due to valence vibration of amidic carbonyl at 1641 cm^−1^. In the case of precursors **5a**,**b**, these two carbonyl absorption peaks are overlapped at 1654 or 1656 cm^−1^. The IR spectrum of *N*-acyl-α-amino acid **3** also showed a strong, broad absorption peak extending from 2500 to 3300 cm^−1^, and three weak bands in the range 2563–2615 cm^−1^ due to the stretching vibration of the O-H bond, which indicates that the molecules of **3** are associated by hydrogen bonds.

The FT-IR spectra of heterocyclic compounds **4** and **6a**,**b** are significantly different from the corresponding spectra of their acyclic intermediates (**3** and **5a**,**b**, respectively) and this proves that cyclizations took place. In the IR spectrum of 1,3-oxazol-5(4*H*)-one **4**, the absorption band due to carbonyl stretching vibration appeared at 1830 cm^−1^, being shifted at a higher wavenumber compared to the corresponding absorption from the precursor **3** spectrum. The IR spectra of **4** and **6a**,**b** showed a band assigned to C = N valence vibration at 1602 (**6b**), 1603 (**6a**), or 1650 cm^−1^ (**4**). Also, a peak due to C-O-C symmetric stretching vibration was observed at 1040 (**4**), 1097 (**6b**), or 1098 cm^−1^ (**6a**), and a band due to C-O-C asymmetric stretching vibration at 1245 (**4**) or 1282 cm^−1^ (**6a,b**).

##### NMR Spectral Data

Proton NMR Spectral Data

**Figure 3:**
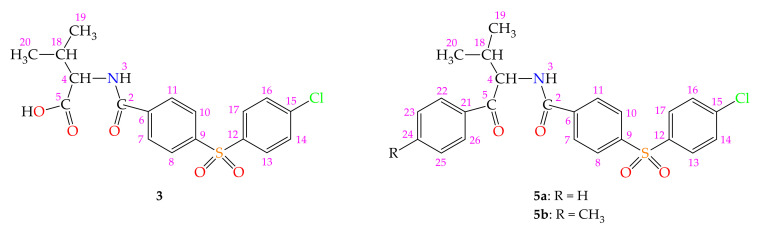
Structures of compounds **3** and **5a**,**b** with the atoms’ numbering (for NMR assignments).

The numbering of atoms used for the assignment of NMR signals of the open-chain precursors **3** and **5a**,**b** is shown in Figure 3.

The ^1^H-NMR spectra of acyclic intermediates **3** and **5a**,**b** presented a signal in the range of 8.73–8.97 ppm, assigned to deshielded NH proton, respectively H-3. The ^1^H-NMR spectrum of *N*-acyl-α-amino acid **3** showed a signal at 4.29 ppm attributed to the H-4, which appeared as a doublet of doublets due to the coupling to protons: H-3 and H-18. In the *N*-acyl-α-amino ketones **5a**,**b** spectra, the triplet signal at 5.36 or 5.38 ppm was assigned to the H-4 proton coupled to the H-3 and H-18 protons. In the ^1^H-NMR spectra of **3** and **5a**,**b**, the isopropyl group was highlighted by the presence of an octet or multiplet signal registered in the 2.18–2.30 ppm region, attributed to the methine proton and two strongly shielded signals as a doublet in the intervals: 0.89–0.94 ppm and 0.92–0.95 ppm, respectively, due to the nonequivalent CH_3_ groups’ protons.

**Figure 4:**
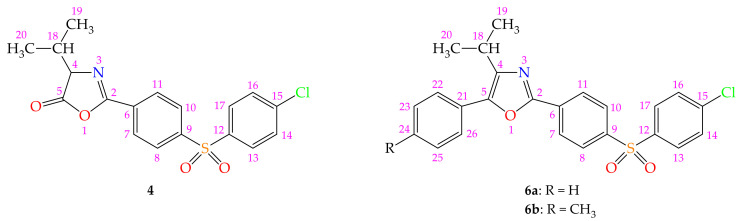
Structures of compounds **4** and **6a**,**b** with the numbering of atoms (used for NMR assignments).

The numbering of atoms used for the assignment of NMR signals of the compounds **4** and **6a**,**b** is presented in Figure 4.

In the ^1^H-NMR spectrum of 1,3-oxazol-5(4*H*)-one **4**, the H-4 signal appeared at 4.32 ppm as a doublet due to the coupling only to the H-18 proton. The H-18 signal was observed at 2.39 ppm as a heptet of doublets due to coupling to the H-19 and H-20 methyl protons (with ^3^*J* = 6.9 Hz) and H-4 proton (with ^3^*J* = 4.7 Hz). The ^1^H-NMR spectra of 1,3-oxazoles **6a**,**b** presented, for the isopropyl group, a heptet signal at 3.26 or 3.29 ppm due to the H-18 proton, and a shielded doublet at 1.35 or 1.36 ppm, due to the protons of the two methyl groups.

Carbon-13 NMR Spectral Data

The ^13^C-NMR spectrum of *N*-acyl-α-amino acid **3** showed a signal at *δ*_C_ = 58.43 ppm due to the C-4 atom, for the isopropyl group a signal at 29.48 ppm due to the presence of the methine carbon and two signals at 18.61 and 19.25 ppm, respectively due to nonequivalent carbon atoms of CH_3_ groups. The C-4 signal was deshielded with 12.61 ppm after cyclization of intermediate **3** to 1,3-oxazol-5(4*H*)-one **4**. Furthermore, the C-2 atom of saturated azlactone **4** resonated at 160.36 ppm, being more shielded with 5.42 ppm than corresponding carbon of **3**, and the C-5 at 176.98 ppm, being shifted downfield with 4.18 ppm compared to the corresponding atom of precursor **3**.

The ^13^C-NMR spectra of 1,3-oxazoles **6a**,**b** presented a signal attributed to C-4 atom at 143.56 or 144.17 ppm, which was more deshielded, with ≈84.73 ppm, than the corresponding carbon signal of *N*-acyl-α-amino ketones **5a**,**b** (from 59.06 or 59.21 ppm). The C-2 signal of heterocycles **6a**,**b** was observed at 157.46 or 157.78 ppm, whereas the corresponding carbon signal of intermediates **5a**,**b** was recorded at 165.49 or 165.56 ppm, highlighting a signal shift for this carbon at smaller *δ*_C_ of about 7.91 ppm. The intramolecular cyclocondensation of *N*-acyl-α-amino ketones **5a**,**b** to compounds **6a**,**b** also induced a shielding effect that results in an approximately 53.35 ppm lower chemical shift for the C-5 atom of the 1,3-oxazole core.

##### Mass Spectral Data

Supplementary support for elucidating the structure of the 1,3-oxazol-5(4*H*)-one **4** was obtained by recording its mass spectrum by GC-EI-MS analysis. Only saturated azlactone **4** could be analyzed by this technique, probably due to its high volatility. Being unstable, the two molecular ions of **4** corresponding to chlorine isotopes (^35^Cl/^37^Cl) did not show peaks in the mass spectrum. They began to fragment by eliminating a molecule of propene, when the base peak with *m/z* = 335 and the corresponding cation-radical with *m*/*z* = 337 (with a relative abundance of 26.69%) were formed in accordance with the ^35^Cl/^37^Cl isotopic ratio of about 3:1. Other main fragments of **4** which occurred under electron impact are reported in the Materials and Methods section, the fragmentation pattern being consistent with the structure of the compound.

### 2.2. *Antimicrobial Activity Assessment*

#### 2.2.1. Qualitative Screening of the Antimicrobial Activity

The results of the qualitative analysis of the antimicrobial activity of the newly synthesized compounds showed that compounds **5a** and **6b** did not produce any growth inhibition zones (Table 1). The compounds **3** and **4** inhibited the growth of the Gram-positive strain *Enterococcus faecium* E5, producing a growth inhibition zone of 15 and 10 mm, respectively. Compound **4** also inhibited the growth of the other two Gram-positive bacteria: *Staphylococcus aureus* ATCC 6538 and *Bacillus subtilis* ATCC 6683, producing a growth inhibition zone of 8 and 9 mm, respectively. The *S. aureus* ATCC 6538 strain was also sensitive to the action of compound **5b**, the growth inhibition diameter being 9 mm. The compound **6a** showed an antimicrobial effect against the *C. albicans* 393 yeast strain, the diameter of growth inhibition being 8 mm. Therefore, the results of the qualitative assay indicate that compound **4** showed a broader spectrum of antimicrobial activity, being active against all studied Gram-positive bacterial strains.

#### 2.2.2. Investigation of the Influence of the Tested Compounds on the Antibiotic Susceptibility Spectrum of the Studied Strains

For compounds **3** and **4** which proved to be the most active in the qualitative disk diffusion assay, their influence on the *E. faecium* E5 and *S. aureus* ATCC 6538 strains’ susceptibility to different antibiotics was evaluated, and the diameters of growth inhibition zones (mm) are shown in Table 2 and Table 3.

No significant changes in the *E. faecium* E5 strain’s susceptibility to current antibiotics were noticed after cultivation in the presence of subinhibitory concentrations of the two tested compounds (Table 2), suggesting both a different mechanism of action and the occurrence of low selective pressure for resistance.

In the case of the strain *S. aureus* ATCC 6538, when grown in the presence of compound **4**, an increase in the area of growth inhibition was observed in the case of azithromycin, penicillin, and cefoxitin (Table 3).

#### 2.2.3. Quantitative Assessment of Antimicrobial Activity

The minimal inhibitory concentration (MIC) and the minimal biofilm eradication concentration (MBEC) values (in µg/mL) obtained for the tested compounds using the microdilution method are presented in Table 4.

The quantitative testing of the antimicrobial activity using the microdilution method showed that the majority of the tested compounds exhibited a low antimicrobial effect, with MIC values equal to or higher than 500 µg/mL. The most active proved to be compound **4** which was found to have moderate antimicrobial activity against two of the tested Gram-positive reference strains, i.e., *Staphylococcus aureus* ATCC 6538 and *Bacillus subtilis* ATCC 6683 (MIC value of 125 µg/mL).

#### 2.2.4. Assessment of the Antibiofilm Activity

Concerning the influence on the development of microbial biofilms on the inert substrate, compounds **5a**, **5b**, and **6b** did not interfere with the development of microbial biofilms on the inert substrate at the tested range of concentrations (Table 4). Compounds **3** and **4** exhibited a moderate antibiofilm effect in the case of the Gram-positive *E. faecium* E5 strain, with an MBEC value of 125 µg/mL. Compound **4** also exhibited an antibiofilm effect against *Staphylococcus aureus* ATCC 6538 (MBEC of 125 µg/mL). Also, compound **6a** showed a moderate antibiofilm activity against *Candida albicans* 393 (MBEC of 125 µg/mL).

### 2.3. *Daphnia Magna Toxicity Assay*

The predicted values for LC_50_ at 48 h range from 0.15 to 3.12 µg/mL, indicating a high toxicity risk for compounds **3**, **4**, **5a**, **5b**, **6a**, and **6b**. However, the experimental results showed that the LC_50_ values are significantly higher than those predicted (Table 5). Thus, compound **5b** induced the highest toxicity, followed by **5a** and then by **6a**, **4**, and **6b**. Compounds **4**, **6a**, and **6b** have a similar toxicity profile, as shown by the 95% CI values, which were similar. Compound **3** showed no toxicity, even at the highest tested concentration. All compounds induced less lethality than control 2, which had an LC_50_ 3-fold lower than compound **5b**.

### 2.4. Prediction of the Molecular Mechanism of Action and Toxicity

#### 2.4.1. PASS Prediction

**Figure 5:**
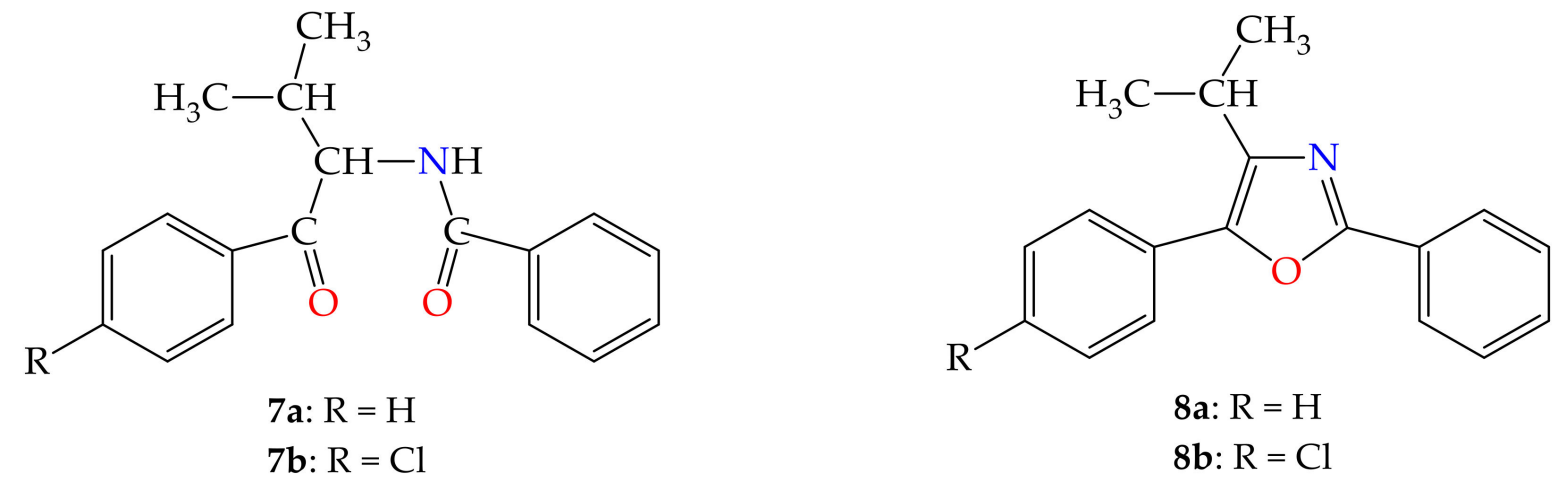
Structures of the designed analogs **7a**,**b** and **8a**,**b**.

Prediction of activity spectra for substances (PASS) is an algorithm that predicts a large panel of biological activities of a given molecule using its structure as input data and yields the probability to be active (Pa) and inactive (Pi), respectively, for each target [74]. The corresponding Pa values obtained for the compounds **3**–**6** are presented in Table 6. In order to test the importance of the 4-chlorobenzenesulfonyl scaffold, we designed the analogs **7a**,**b** and **8a**,**b**, whose structures are shown in Figure 5, and whose Pa values are presented in Table 6.

The Pa values are an indication of the possibility that the new compounds produce an effect, but not for the potency of their biological activity. The Pa values are generally higher for compounds **5a** and **5b** than for the corresponding 1,3-oxazoles **6a** and **6b**. The comparison of the Pa values of the compounds **5a**,**b** with the related compounds **7a**,**b** and the Pa values for **6a**,**b** and those of **8a**,**b**, respectively, indicates the favorable role of the 4-chlorobenzenesulfonyl fragment for antimicrobial potential.

#### 2.4.2. Structural Similarity Analysis

**Figure 6:**
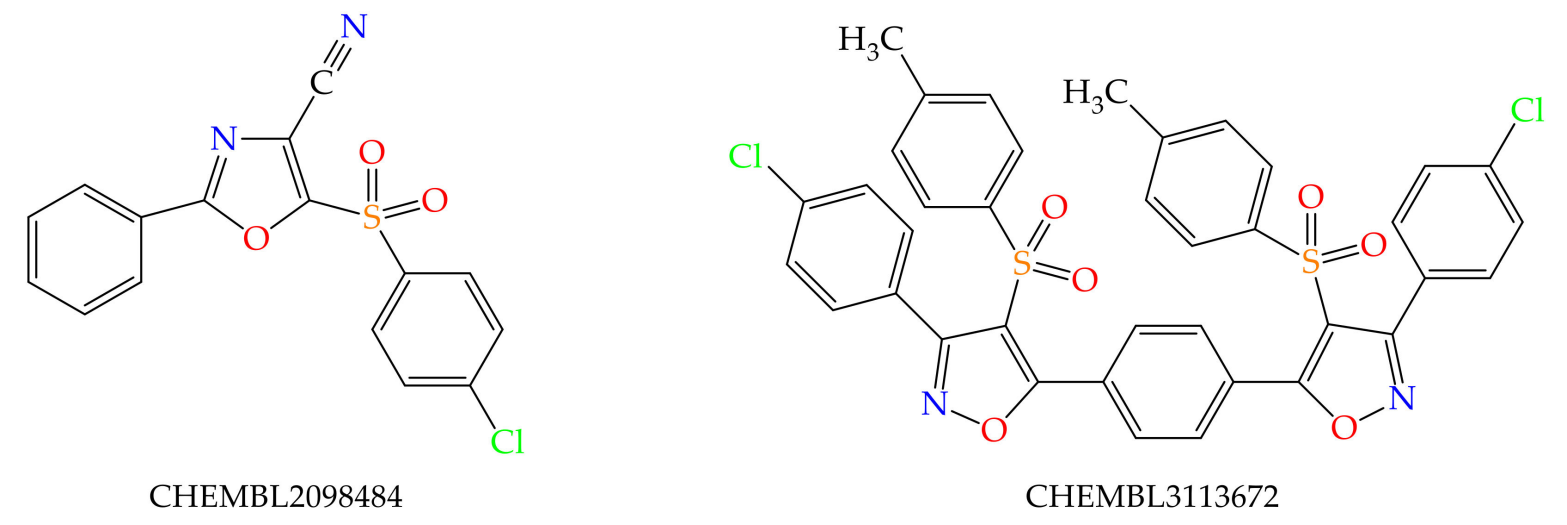
Structures of two compounds extracted from ChEMBL which are structurally similar to the compounds **6a** and **6b**.

The similarity search on the ChEMBL database for the newly synthesized compounds **3**, **4**, **5a**,**b**, and **6a**,**b** returned 60 analog compounds, with the highest degree of structural similarity (82.50%) being observed for the pair formed by compound CHEMBL2071494 and **5a**. The results highlight the originality of the newly synthesized compounds. The success of this type of similarity analysis depends on the volume of data on the resulting compounds. For only 2 of the 60 compounds, there are antimicrobial results available (Figure 6).

Compound CHEMBL2098484 has a 50.9% structural similarity with **6a** and demonstrated antimicrobial activities on *Mycobacterium tuberculosis*, with MIC values between 6.25 and 12.5 μM depending on the experimental conditions and the bacterial strain. The compound presented low inhibition effects on several bacterial pathogens: *Escherichia coli* (6.5%), *Klebsiella pneumoniae* (−0.9%), *Acinetobacter baumannii* (2.8%), *Pseudomonas aeruginosa* (0.8%), and *Staphylococcus aureus* MRSA ATCC 43300 (8.6%) at a concentration of 32 μg/mL.

CHEMBL3113672, a (1,4-phenylene)bis(arylsufonylisoxazoles) derivative, has a 50% structural similarity with **6b** and potent antimicrobial effect with MIC values of 25 μg/mL on *Bacillus subtilis*, 50 μg/mL on *Staphylococcus aureus* and *Pseudomonas aeruginosa*, 100 μg/mL on *Klebsiella pneumoniae*.

## 3. Discussion

1,3-Oxazoles are a vast class of five-membered heterocyclic aromatic compounds containing a nitrogen atom in 1-position and an oxygen atom in 3-position as part of the ring and having two double bonds. It is well known in the literature that many compounds containing a 1,3-oxazole motif in their structure are active ingredients in modern antimicrobial, anti-inflammatory, antitumor, and antidiabetic drugs. Thus, they are promising candidates for the development of new antimicrobial agents active against drug-resistant bacterial and fungal strains. In recent years, 1,3-oxazole clubbed pyridyl-pyrazolines with antimicrobial activity [75], steroidal oxazole derivatives with effective antimicrobial and antibiofilm properties [76], derivatives of pyrazolecarboxamide containing oxazole ring displaying good antifungal activity [13], substituted oxazole-benzamides as antibacterial inhibitors of FtsZ [18], and propanoic acid derivatives incorporating oxazole core with antibacterial and antifungal activity [77], etc, have been reported.

Given the biological importance of the final products of this work, namely of heterocyclic compounds from the class of 1,3-oxazoles, these compounds were obtained over time by a variety of synthetic methods [9]. Our multi-step synthesis strategy used a natural α-amino acid as the starting material and led to good yields of the newly synthesized compounds of the four chemical classes (*N*-acyl-α-amino acids, 1,3-oxazol-5(4*H*)-ones, *N*-acyl-α-amino ketones, 1,3-oxazoles), which were further characterized by physico-chemical methods and assessed for their antimicrobial and antibiofilm effects, toxicity on *D. magna*, and by in silico studies to predict their potential mechanism of action and toxicity. The biological activity data showed that compounds **3** and **4** have a moderate antibacterial activity against Gram-positive bacterial strains and **6a** against *C. albicans* strain. *D. magna*, assay revealed that the newly synthesized compounds induced moderate to high toxicity, with one of them being non-toxic. Despite the variation in toxicity of the compounds; from high for *N*-acyl-α-amino ketones **5a** and **5b**, to medium for 1,3-oxazol-5(4*H*)-one **4**, 1,3-oxazoles **6a** and **6b**, and to non-toxic for *N*-acyl-α-amino acid **3**, all compounds showed significantly lower toxicity than compound **1**, which is a starting material. Thus, through this synthesis, the toxicity of the newly synthesized compounds was decreased significantly.

It is worth mentioning that the *N*-acyl-α-amino acid **3** exhibited promising antibacterial and antibiofilm activities, without toxicity. It can be seen that the transformation of compound **3** to the corresponding saturated azlactone **4** resulted in an improvement of the antimicrobial and antibiofilm profiles, but with an increase in toxicity. In the range of tested concentrations, the *N*-acyl-α-amino ketone **5a** was proved inactive against the studied strains, but by cyclodehydration, the corresponding 1,3-oxazole **6a** was obtained, showing an antibiofilm effect in the case of the pathogenic yeast *C. albicans* 393. Furthermore, it appears that the conversion of 1,3-oxazol-5(4*H*)-one **4** to *N*-acyl-α-amino ketone **5b** leads to a decrease in antibacterial and antibiofilm activities, as well as by cyclization of **5b** to 1,3-oxazole **6b**. The biological action of compounds **3**, **4**, and **6a** is probably due to the presence in their structure of the valine residue, the 4-[(4-chlorophenyl)sulfonyl]phenyl fragment, and the other structural features. The synthesis of the scaffold of final products **6a,b**, without R, Cl, and isopropyl functional groups, namely of the 5-phenyl-2-[4-(phenylsulfonyl)phenyl]-1,3-oxazole has been reported by Schiketanz et al. in the early 2000s [33], but this non-substituted prototype has not been tested for antimicrobial activity. It is not clear whether the substituents, especially the isopropyl, are critical for the reported antibacterial effect, but in future works, we will approach this topic as well.

Also, in the future, we will consider some structural optimizations with the purpose of obtaining new potent agents. In this regard, we identified three critical positions on the molecular scaffolds which can influence the biological effect. A first choice for the future improvement of these derivatives is to replace the chlorine atom from the arylsulfonylphenyl moiety with various substituents, e.g., an iodine atom, trifluoromethyl, or nitro groups. It was observed that the introduction of an electron-withdrawing group (such as NO_2_) on aromatic rings increased the antibacterial potency of the compounds [78]. Furthermore, it was demonstrated that the iodine atom is more active than the other halogens, multiple-halogen-substituted derivatives displayed best antibacterial activity than their respective mono-halogen-substituted derivatives, hydrophobic groups (e.g., *tert*-butyl, benzyloxy) linked to the aryl moiety deliver prominent antibacterial activity [77]. It was also shown that small substituents (like hydroxy group) at the 4-position of the phenyl ring improved the antibacterial potency [18]. For this goal, we can use as raw materials other commercially available aromatic compounds in the Friedel–Crafts sulfonylation. As a second possibility of optimization, other α-amino acids (e.g., leucine, isoleucine, methionine) will be used in Steiger acylation with acyl chloride **2** or we will consider the possibility of including more α-amino acid residues in molecules because it is known that naturally occurring oxazole-containing peptides, like microcin B17, are DNA-gyrase inhibitors [4]. A third choice for improving the antimicrobial efficacy and antibiofilm effect is to use other benzene-derived compounds with various substituents (such as an iodine atom or a trifluoromethyl group) in the acylation reaction with 1,3-oxazol-5(4*H*)-one **4**. The in silico studies showed that the most probable pharmacological targets for 1,3-oxazoles **6a** and **6b** are: CHEMBL2098484 and CHEMBL3113672, respectively, highlighting a potential future synthesis approach.

## 4. Materials and Methods

### 4.1. General Information

The melting points, m.p., were determined on a Boëtius hot plate microscope (VEB Wägetechnik Rapido, PHMK 81/3026, Radebeul, Germany) and are uncorrected. The UV-Vis spectra were recorded for solutions in methanol (≈0.025 mM) on a Specord 40 spectrophotometer (Analytik Jena AG, Jena, Germany) using a quartz cuvette with a path length of 1 cm. The FT-IR spectra were measured on a Vertex 70 spectrometer (Bruker Optik GmbH, Ettlingen, Germany) as KBr pellets. The intensities of selected IR bands are given as vs, very strong; s, strong; m, medium; w, weak. The NMR spectra were registered on a Gemini 300 BB spectrometer (Varian, Inc., Palo Alto, CA, USA) operating at 300 MHz for ^1^H and 75 MHz for ^13^C in deuterated solvents (DMSO-*d*_6_ or CDCl_3_) at room temperature. Supplementary evidence was obtained by the 2D HETCOR experiment. The chemical shifts were recorded as *δ* values in parts per million (ppm) relative to tetramethylsilane (TMS) and coupling constants *(J*) are reported in hertz (Hz). The splitting patterns are abbreviated as follows: s, singlet; d, doublet; dd, doublet of doublets; t, triplet; tt, triplet of triplets; hp, heptet; hpd, heptet of doublets; oct, octet; m, multiplet; a broad signal is abbreviated br. ^1^H-NMR data are reported in the following order: chemical shift (multiplicity, coupling constants, number of protons, proton assignment) and ^13^C-NMR data are cited as follows: chemical shift (carbon assignment). The mass spectrum of **4** was recorded on a GC 8000 gas chromatograph (with an electron impact quadrupole) coupled to an MD 800 mass spectrometer detector (Fisons Instruments SpA, Rodano, Milan, Italy), in dichloromethane as the solvent, using an SLB-5ms capillary column (30 m × 0.32 mm, d_f_ 0.25 μm); flow rate of carrier gas (helium) was 2 mL/min. RP-HPLC was carried out on a System Gold 126 liquid chromatograph (Beckman Coulter, Inc., Fullerton, CA, USA), with a System Gold 166 UV-Vis detector, a Rheodyne injection system, and a LiChrosorb RP-18 column (25 cm × 4.6 mm, 5 μm particle size), using a flow rate of mobile phase (a methanol–water mixture in various volume ratios) of 1 mL/min. Purity of compounds (%) and retention time, *t*_R_, in minutes (min) were reported. The elemental analysis was performed on an ECS 4010 elemental combustion system (Costech Analytical Technologies Inc., Valencia, CA, USA).

### 4.2. Chemistry

Chemicals and reagents were obtained from commercially available suppliers and used without purification. The dichloromethane was dried over anhydrous CaCl_2_.

#### 4.2.1. Synthesis of 2-{4-[(4-Chlorophenyl)sulfonyl]benzamido}-3-methylbutanoic Acid **3**

Valine (2.34 g, 20 mmol) was dissolved in 20 mL (20 mmol) of 1 N NaOH solution. To this solution, cooled in an ice bath (0–5 °C), a solution of raw 4-[(4-chlorophenyl)sulfonyl]benzoyl chloride **2** (6.30 g, 20 mmol) in 45 mL of anhydrous CH_2_Cl_2_, and a 2 N NaOH solution (10 mL, 20 mmol), respectively, were added simultaneously, dropwise, under magnetic stirring, for 30 min. Stirring of the reaction mixture was continued for another 1 h at room temperature, then, the aqueous phase was separated and acidified with 2 N HCl. The precipitated solid was filtered off, washed with water, dried, and recrystallized from water, when white acicular crystals were obtained; yield = 93% (7.36 g); m.p. = 191–193 °C.

UV-Vis (CH_3_OH, *λ* nm) (lg *ε*): 202.6 (4.48); 248.5 (4.08).

FT-IR (KBr, *ν* cm^−1^): 3358 s; 3090 m; 3074 m; 2964 s; 2937 m; 2876 m; 2615 w; 2577 w; 2563 w; 1737 vs; 1641 vs; 1601 m; 1555 s; 1478 m; 1468 m; 1320 vs; 1303 s; 1290 s; 1283 s; 1160 vs; 849 s; 759 vs.

^1^H-NMR (DMSO-*d*_6_, *δ* ppm, *J* Hz): 0.94 (d, 6.9, 3H, H-19); 0.95 (d, 6.9, 3H, H-20); 2.18 (oct, 6.9, 1H, H-18); 4.29 (dd, 8.1, 6.9, 1H, H-4); 7.71 (d, 8.5, 2H, H-14, H-16); 8.00 (d, 8.5, 2H, H-13, H-17); 8.05 (d, 8.8, 2H, H-8, H-10); 8.09 (d, 8.8, 2H, H-7, H-11); 8.73 (d, 8.0, 1H, H-3).

^13^C-NMR (DMSO-*d*_6_, *δ* ppm): 18.61 (C-19); 19.25 (C-20); 29.48 (C-18); 58.43 (C-4); 127.49 (C-8, C-10); 129.08 (C-13, C-17); 129.46 (C-7, C-11); 130.01 (C-14, C-16); 139.03 (C-6); 139.10 (C-15); 139.51 (C-12); 142.70 (C-9); 165.78 (C-2); 172.80 (C-5).

RP-HPLC (methanol/water 30:70, *v*/*v*; 1 mL/min; 250 nm): purity = 99.99%; *t*_R_ = 4.38 min.

Elemental analysis (%): calculated for C_18_H_18_ClNO_5_S (395.86 g/mol): C, 54.61; H, 4.58; N, 3.54; S, 8.10 and found: C, 54.66; H, 4.57; N, 3.54; S, 8.13.

#### 4.2.2. Synthesis of 2-{4-[(4-Chlorophenyl)sulfonyl]phenyl}-4-isopropyl-1,3-oxazol-5(4*H*)-one **4**

2-{4-[(4-Chlorophenyl)sulfonyl]benzamido}-3-methylbutanoic acid **3** (4.16 g, 10.5 mmol) was suspended in anhydrous dichloromethane (50 mL), and then 1.15 mL (10.5 mmol) of 4-methylmorpholine was added under magnetic stirring, at room temperature. An equimolar quantity of ethyl chloroformate (1 mL, 10.5 mmol) was added in drops under stirring to the previously formed solution. The reaction mixture was stirred for another 30 min at room temperature and then poured over 100 mL of ice water. The organic phase was separated and washed with 5% NaHCO_3_ solution, then with water, and dried (MgSO_4_). After vacuum concentration and recrystallization (from cyclohexane), the compound **4** was obtained as white crystals; yield = 93% (3.69 g); m.p. = 139–141 °C.

UV-Vis (CH_3_OH, *λ* nm) (lg *ε*): 202.6 (4.48); 222.9 (4.14); 249.3 (4.32).

FT-IR (KBr, *ν* cm^−1^): 3098 w; 3072 w; 3048 w; 2968 m; 2933 m; 2876 w; 1830 vs; 1650 vs; 1598 m; 1579 m; 1476 m; 1328 vs; 1289 s; 1245 m; 1158 vs; 1040 vs; 847 m; 768 vs.

^1^H-NMR (CDCl_3_, *δ* ppm, *J* Hz): 0.99 (d, 6.9, 3H, H-19); 1.14 (d, 6.9, 3H, H-20); 2.39 (hpd, 6.9, 4.7, 1H, H-18); 4.32 (d, 4.7, 1H, H-4); 7.51 (d, 8.5, 2H, H-14, H-16); 7.91 (d, 8.5, 2H, H-13, H-17); 8.05 (d, 8.5, 2H, H-8, H-10); 8.16 (d, 8.5, 2H, H-7, H-11).

^13^C-NMR (CDCl_3_, *δ* ppm): 17.65 (C-19); 18.89 (C-20); 31.42 (C-18); 71.04 (C-4); 128.18 (C-8, C-10); 128.98 (C-7, C-11); 129.44 (C-13, C-17); 129.98 (C-14, C-16); 130.57 (C-6); 139.38 (C-15); 140.63 (C-12); 144.95 (C-9); 160.36 (C-2); 176.98 (C-5).

GC-EI-MS (*m*/*z*, rel. abund. %): 335 (^35^Cl)/337 (^37^Cl) (100, BP/26.69) [M-C_3_H_6_]^+^^⋅^; 279 (43.22) [^35^ClC_6_H_4_SO_2_C_6_H_4_CHNH]^+^^⋅^ or [^35^ClC_6_H_4_SO_2_C_6_H_4_CO]^+^; 280 (18.22) [^37^ClC_6_H_4_SO_2_C_6_H_4_CNH]^+^; 252 (16.31) [^35^ClC_6_H_4_SO_2_C_6_H_5_]^+^^⋅^; 159/161 (44.07/13.14) [^35^ClC_6_H_4_SO]^+^/[^37^ClC_6_H_4_SO]^+^; 131/133 (7.63/5.30); 111 (13.98) [^35^ClC_6_H_4_]^+^; 44 (18.22) [C_3_H_8_]^+^^⋅^ or [CO_2_]^+^^⋅^; 43 (34.53) [C_3_H_7_]^+^; *t*_R_ = 31.30 min.

RP-HPLC (methanol/water 60:40, *v*/*v*; 1 mL/min; 250 nm): purity = 96.28%; *t*_R_ = 3.93 min.

Elemental analysis (%): calculated for C_18_H_16_ClNO_4_S (377.84 g/mol): C, 57.22; H, 4.27; N, 3.71; S, 8.49 and found: C, 57.17; H, 4.26; N, 3.71; S, 8.46.

#### 4.2.3. General Procedure for the Synthesis of the *N*-(1-Aryl-3-methyl-1-oxobutan-2-yl)-4-[(4-chlorophenyl)sulfonyl]benzamides **5a**,**b**

To a solution of raw 2-{4-[(4-chlorophenyl)sulfonyl]phenyl}-4-isopropyl-1,3-oxazol-5(4*H*)-one **4** (1.72 g, 5 mmol) in 25 mL of anhydrous aromatic hydrocarbon (benzene or toluene), 2.00 g (15 mmol) of anhydrous AlCl_3_ were added in portions, under magnetic stirring, at room temperature. The reaction mixture was stirred for 20 h until HCl emission ceased and then poured over 100 mL of ice water with 5 mL of 37% HCl. The formed solid was filtered off, washed with cold water, and further with a cold ethanol–water mixture (1:1, *v*/*v*). The aqueous filtrate was extracted with CH_2_Cl_2_ (2 × 15 mL). The organic phase was washed with water, dried (Na_2_SO_4_), and concentrated by vacuum distillation, when the second fraction of crude product was obtained. Purification by recrystallization from ethanol yielded product **5** as colorless crystals.

##### 4-[(4-Chlorophenyl)sulfonyl]-*N*-(3-methyl-1-oxo-1-phenylbutan-2-yl)benzamide **5a**

*N*-Acyl-α-amino ketone **5a** was prepared by reaction of compound **4** with benzene (21.91 g, 280 mmol).

Yield = 86% (1.96 g). m.p. = 179–181 °C.

UV-Vis (CH_3_OH, *λ* nm) (lg *ε*): 202.6 (4.49); 249.3 (4.17).

FT-IR (KBr, *ν* cm^−1^): 3299 s; 3091 w; 3061 w; 3039 w; 2959 m; 2933 w; 2872 w; 1654 vs; 1596 m; 1579 s; 1529 vs; 1478 m; 1448 m; 1322 vs; 1298 s; 1287 s; 1163 vs; 856 m; 758 vs.

^1^H-NMR (DMSO-*d*_6_, *δ* ppm, *J* Hz): 0.92 (d, 6.6, 3H, H-19); 0.94 (d, 6.6, 3H, H-20); 2.30 (m, 1H, H-18); 5.38 (t, 7.4, 1H, H-4); 7.53 (t, 7.4, 2H, H-23, H-25); 7.64 (tt, 7.4, 1.4, 1H, H-24); 7.69 (d, 8.5, 2H, H-14, H-16); 7.98 (d, 8.5, 2H, H-13, H-17); 8.04 (m, 6H, H-7, H-8, H-10, H-11, H-22, H-26); 8.88 (brs, 1H, H-3).

^13^C-NMR (DMSO-*d_6_*, *δ* ppm): 18.35 (C-19); 19.71 (C-20); 29.49 (C-18); 59.21 (C-4); 127.52 (C-8, C-10); 128.23 (C-22, C-26); 128.81 (C-23, C-25); 129.02 (C-7, C-11); 129.45 (C-13, C-17); 129.99 (C-14, C-16); 133.43 (C-24); 136.16 (C-21); 138.77 (C-6); 139.09 (C-15); 139.45 (C-12); 142.79 (C-9); 165.56 (C-2); 199.18 (C-5).

RP-HPLC (methanol/water 60:40, *v*/*v*; 1 mL/min; 250 nm): purity = 98.60%; *t*_R_ = 4.47 min.

Elemental analysis (%): calculated for C_24_H_22_ClNO_4_S (455.95 g/mol): C, 63.22; H, 4.86; N, 3.07; S, 7.03 and found: C, 63.25; H, 4.84; N, 3.06; S, 7.05.

##### 4-[(4-Chlorophenyl)sulfonyl]-*N*-[3-methyl-1-oxo-1-(*p*-tolyl)butan-2-yl]benzamide **5b**

*N*-Acyl-α-amino ketone **5b** was prepared by reaction of compound **4** with toluene (21.63 g, 234.75 mmol).

Yield = 90% (2.11 g). m.p. = 147–149 °C.

UV-Vis (CH_3_OH, *λ* nm) (lg *ε*): 202.6 (4.48); 252.9 (4.21).

FT-IR (KBr, *ν* cm^−1^): 3279 s; 3087 m; 3058 w; 3037 m; 2963 m; 2931 m; 2869 m; 1656 vs; 1606 s; 1574 s; 1530 vs; 1478 s; 1467 m; 1326 vs; 1305 s; 1286 s; 1160 vs; 843 m; 756 vs.

^1^H-NMR (DMSO-*d*_6_, *δ* ppm, *J* Hz): 0.89 (d, 7.1, 3H, H-19); 0.92 (d, 7.1, 3H, H-20); 2.27 (m, 1H, H-18); 2.35 (s, 3H, CH_3_); 5.36 (t, 7.7, 1H, H-4); 7.33 (d, 8.4, 2H, H-23, H-25); 7.70 (d, 8.8, 2H, H-14, H-16); 8.00 (m, 4H, H-13, H-17, H-22, H-26); 8.03 (d, 8.8, 2H, H-8, H-10); 8.07 (d, 8.8, 2H, H-7, H-11); 8.97 (d, 8.1, 1H, H-3).

^13^C-NMR (DMSO-*d*_6_, *δ* ppm): 18.36 (C-19); 19.73 (C-20); 21.15 (CH_3_); 29.59 (C-18); 59.06 (C-4); 127.53 (C-8, C-10); 128.40 (C-22, C-26); 129.04 (C-7, C-11); 129.37 (C-23, C-25); 129.46 (C-13, C-17); 130.00 (C-14, C-16); 133.63 (C-21); 138.81 (C-6); 139.11 (C-15); 139.48 (C-12); 142.78 (C-9); 143.93 (C-24); 165.49 (C-2); 198.60 (C-5).

RP-HPLC (methanol–water 60:40, *v*/*v*; 1 mL/min; 250 nm): purity = 97.51%; *t*_R_ = 4.98 min.

Elemental analysis (%): calculated for C_25_H_24_ClNO_4_S (469.98 g/mol): C, 63.89; H, 5.15; N, 2.98; S, 6.82 and found: C, 63.94; H, 5.13; N, 2.97; S, 6.85.

#### 4.2.4. General Procedure for the Synthesis of the 5-Aryl-2-{4-[(4-chlorophenyl)sulfonyl]phenyl}-4-isopropyl-1,3-oxazoles **6a**,**b**

Crude *N*-(1-aryl-3-methyl-1-oxobutan-2-yl)-4-[(4-chlorophenyl)sulfonyl]benzamide **5** (10 mmol) in phosphoryl trichloride (20 mL, 217.83 mmol) was heated under reflux for 4 h. Phosphoryl trichloride excess was removed by distillation in vacuo. The oily residue was slowly poured onto crushed ice and extracted with CH_2_Cl_2_ (2 × 20 mL). The organic phase was separated and washed with 5% NaHCO_3_ solution, then with water, and dried over anhydrous Na_2_SO_4_. The solvent was concentrated under reduced pressure and then the raw solid was recrystallized from ethanol when colorless crystals were obtained.

##### 2-{4-[(4-Chlorophenyl)sulfonyl]phenyl}-4-isopropyl-5-phenyl-1,3-oxazole **6a**

1,3-Oxazole **6a** was prepared from 4.56 g of 4-[(4-chlorophenyl)sulfonyl]-*N*-(3-methyl-1-oxo-1-phenylbutan-2-yl)benzamide **5a**.

Yield = 90% (3.94 g). m.p. = 169–171 °C.

UV-Vis (CH_3_OH, *λ* nm) (lg *ε*): 202.6 (4.49); 248.5 (4.11); 333.9 (4.04).

FT-IR (KBr, *ν* cm^−1^): 3094 m; 3063 w; 3042 w; 2964 m; 2931 m; 2870 m; 1603 m; 1586 m; 1544 w; 1494 m; 1477 m; 1446 m; 1323 s; 1292 m; 1282 m; 1157 vs; 1098 s; 847 m; 769 s.

^1^H-NMR (CDCl_3_, *δ* ppm, *J* Hz): 1.36 (d, 6.9, 6H, H-19, H-20); 3.29 (hp, 6.9, 1H, H-18); 7.37 (tt, 7.4, 1.4, 1H, H-24); 7.48 (brt, 7.4, 2H, H-23, H-25); 7.49 (d, 8.8, 2H, H-14, H-16); 7.65 (dd, 7.4, 1.4, 2H, H-22, H-26); 7.90 (d, 8.8, 2H, H-13, H-17); 8.01 (d, 8.8, 2H, H-8, H-10); 8.22 (d, 8.8, 2H, H-7, H-11).

^13^C-NMR (CDCl_3_, *δ* ppm): 22.05 (C-19, C-20); 26.08 (C-18); 126.24 (C-22, C-26); 127.08 (C-8, C-10); 128.25 (C-7, C-11); 128.38 (C-24); 128.81 (C-21); 129.00 (C-23, C-25); 129.22 (C-13, C-17); 129.81 (C-14, C-16); 132.30 (C-6); 140.01 (C-15); 140.20 (C-9); 141.76 (C-12); 144.17 (C-4); 145.46 (C-5); 157.78 (C-2).

RP-HPLC (methanol/water 70:30, *v*/*v*; 1 mL/min; 335 nm): purity = 97.72%; *t*_R_ = 5.57 min.

Elemental analysis (%): calculated for C_24_H_20_ClNO_3_S (437.94 g/mol): C, 65.82; H, 4.60; N, 3.20; S, 7.32 and found: C, 65.87; H, 4.58; N, 3.20; S, 7.35.

##### 2-{4-[(4-Chlorophenyl)sulfonyl]phenyl}-4-isopropyl-5-(*p*-tolyl)-1,3-oxazole **6b**

1,3-Oxazole **6b** was prepared from 4.70 g of 4-[(4-chlorophenyl)sulfonyl]-*N*-[3-methyl-1-oxo-1-(*p*-tolyl)butan-2-yl]benzamide **5b**.

Yield = 91% (4.11 g). m.p. = 215–217 °C.

UV-Vis (CH_3_OH, *λ* nm) (lg *ε*): 202.6 (4.48); 248.5 (4.10); 337.4 (4.12).

FT-IR (KBr, *ν* cm^−1^): 3095 m; 3072 w; 3029 w; 2963 m; 2926 m; 2870 m; 1602 m; 1590 m; 1544 w; 1508 m; 1478 m; 1322 s; 1296 m; 1282 m; 1155 vs; 1097 s; 847 m; 773 s.

^1^H-NMR (CDCl_3_, *δ* ppm, *J* Hz): 1.35 (d, 6.9, 6H, H-19, H-20); 2.41 (s, 3H, CH_3_); 3.26 (hp, 6.9, 1H, H-18); 7.28 (d, 8.8, 2H, H-23, H-25); 7.49 (d, 8.8, 2H, H-14, H-16); 7.53 (d, 8.2, 2H, H-22, H-26); 7.90 (d, 8.8, 2H, H-13, H-17); 8.00 (d, 8.8, 2H, H-8, H-10); 8.21 (d, 8.8, 2H, H-7, H-11).

^13^C-NMR (CDCl_3_, *δ* ppm): 21.40 (CH_3_); 22.01 (C-19, C-20); 26.01 (C-18); 126.15 (C-22, C-26); 126.96 (C-8, C-10); 128.18 (C-7, C-11, C-24); 129.16 (C-23, C-25); 129.63 (C-13, C-17); 129.76 (C-14, C-16); 132.35 (C-6); 138.42 (C-21); 140.00 (C-15); 140.13 (C-9); 141.56 (C-12); 143.56 (C-4); 145.62 (C-5); 157.46 (C-2).

RP-HPLC (methanol/water 70:30, *v*/*v*; 1 mL/min; 335 nm): purity = 99.99%; *t*_R_ = 6.42 min.

Elemental analysis (%): calculated for C_25_H_22_ClNO_3_S (451.97 g/mol): C, 66.44; H, 4.91; N, 3.10; S, 7.09 and found: C, 66.40; H, 4.89; N, 3.11; S, 7.07.

### 4.3. Antimicrobial Activity Assessment

The antimicrobial activity of the tested compounds was investigated using the agar disc diffusion method, broth microdilution, and microtiter plate test.

#### 4.3.1. Microbial Strains

The study of the antimicrobial activity of the newly synthesized compounds was performed using Gram-positive microbial strains: *E. faecium* E5, *S. aureus* ATCC 6538, *B. subtilis* ATCC 6683, Gram negative bacteria: *P. aeruginosa* ATCC 27857, *E. coli* ATCC 8739, and a yeast strain represented by *C. albicans* 393.

#### 4.3.2. Qualitative Evaluation of Antimicrobial Activity

The qualitative screening of the sensitivity of different microbial strains to the new compounds was achieved by the adapted disc diffusion method, consisting in the distribution of a volume of 5 µL from the compound solution directly on the Mueller-Hinton medium previously seeded with a standardized microbial suspension. The bacterial inoculum was represented by a suspension in sterile physiological water with a microbial density of 0.5 McFarland made from 24 h cultures grown on a nonselective agar medium. The compound stock solution was performed in DMSO (concentration of 5 mg/mL). Ciprofloxacin (5 μg, Oxoid) and fluconazole (25 μg, Oxoid) served as positive controls. The reading of the results was performed after 24 h of incubation at 37 °C by measuring the diameters of the growth inhibition zones generated by the presence of tested substances. The DMSO solvent was comparatively tested for its potential antimicrobial activity.

#### 4.3.3. Investigation of the Influence of the Tested Compounds on the Antibiotic Susceptibility Spectrum of the Studied Strains

Subinhibitory concentrations of tested compounds **3** and **4** were obtained in a sterile liquid culture medium which was then inoculated with 0.5 McFarland suspensions achieved from the 24 h microbial culture of *E. faecium* E5 (**3** and **4**) and *S. aureus* ATCC 6538 (**4**), respectively. A control of DMSO (bacterial strain grown in the presence of DMSO) and control of microbial growth (culture medium inoculated with microbial suspension) were also prepared. The inoculated tubes were incubated at 37 °C for 24 h. The microbial cultures obtained in the presence of subinhibitory concentrations of tested compounds **3** and **4**, DMSO control and microbial growth control cultures, respectively, were used to determine the influence of the tested compounds on the antibiotic susceptibility spectrum of the studied strains. The liquid microbial cultures were sedimented by centrifugation at 10,000 *g* for 5 min, and the obtained cellular sediment was washed 3 times in sterile saline by centrifugation at 10,000 *g* for 5 min. The cell pellet was resuspended in sterile saline until a turbidity corresponding to the 0.5 McFarland standard was obtained and the classical Kirby-Bauer method was then performed to assess the susceptibility to the following antibiotics (bioMérieux, France): ampicillin, penicillin, linezolid, vancomycin for the *E. faecium* E5 strain and azithromycin, penicillin, vancomycin, linezolid, trimethoprim-sulfamethoxazole, clindamycin, rifampicin, and cefoxitin for the *S. aureus* ATCC 6538 strain, respectively. The results were recorded after 24 h of incubation at 37 °C. The diameters of the areas of inhibition of bacterial growth were interpreted according to the recommendations of the current edition of the *Clinical and Laboratory Standards Institute* (CLSI).

#### 4.3.4. Quantitative Testing of the Antimicrobial Activity

The quantitative screening was performed by the method of serial microdilutions in liquid medium (Mueller-Hinton, Oxoid Ltd., Hampshire, UK) using 96-well microtiter plates in order to determine the minimum inhibitory concentration (MIC), which is the minimum amount of tested compound capable of inhibiting microbial cell growth. In a volume of 100 μL of the medium, binary serial dilutions of the stock solution of the compound made in DMSO (0.5 mg/mL) were performed. The tested concentrations of the solutions of the different compounds in DMSO achieved through double serial dilutions were between 500–0.97 µg/mL. Then, the wells were inoculated with 10 µL microbial suspension prepared in the same medium after dilution (1:100) of standardized microbial inoculum adjusted to 0.5 McFarland scale. Culture (wells containing 10 µL of standardized inoculum and 90 μL of Mueller Hinton Broth) and sterility (wells containing 100 µL of Mueller Hinton Broth) controls have been used. Ciprofloxacin (Sigma-Aldrich, St. Louis, MO, USA) and fluconazole (Sigma Aldrich) served as positive controls. The microtiter plates were incubated without agitation for 24 h at 37 °C. In order to confirm the MIC value, the assays were performed in triplicate. The MIC was determined as the lowest concentration of tested compound that inhibited the growth of the microorganism as detected spectrophotometrically at 620 nm with an Apollo LB 911 ELISA Reader (Berthold Technologies GmbH & Co. KG, Waltham, MA, USA) [79].

#### 4.3.5. Evaluation of the Antibiofilm Activity

The assessment of the antibiofilm activity of the tested compounds was carried out using the microtiter biofilm inhibition assay. Briefly, serial two-fold dilutions of the tested compounds were obtained in a 96-well polystyrene microtiter plate which was further inoculated with standard microbial suspensions, using microbial culture and sterility controls, similar to the MIC assay. Ciprofloxacin (Sigma-Aldrich) and fluconazole (Sigma-Aldrich) served as positive controls. The microplates were incubated for 24 h at 37 °C under static conditions to allow for microbial adherence and biofilm development. The wells of the microplate were emptied and washed twice with phosphate-buffered saline. The biofilms formed on the walls of wells of the microplate were fixed with 80% methanol for 5 min and stained with 1% crystal violet solution for 15 min and then rinsed three times with distilled water to remove the unbound dye. The fixed dye was resuspended in 33% acetic acid and the absorbance was measured at the wavelength of 492 nm with an Apollo LB 911 ELISA Reader. The minimal biofilm eradication concentration (MBEC) was determined to be the lowest concentration of the tested compounds at which the decrease in absorbance value, recorded at 492 nm, was observed in comparison to the positive control. Results from at least three separate biological replicates were averaged [80].

### 4.4. *Daphnia Magna Toxicity Assay*

*D. magna* Straus was maintained parthenogenetically (‘Carol Davila’ University—Department of Pharmaceutical Botany and Cell Biology) at 25 °C with a photoperiod of 16 h/8 h light/dark cycle in a Sanyo MLR-351 H climatic chamber (Sanyo, San Diego, CA, USA). For the assay, young daphnids were selected according to their size and maintained for 24 h in an artificial medium. Each compound was tested in six concentrations, chosen according to a pre-screening test and the compounds’ solubilities. A 1% DMSO solution as a negative control. For each sample, 10 daphnids were used per replicate and the determination was carried out in duplicate [81,82,83]. The lethality was evaluated after 24 and 48 h of exposure. LC_50_ and 95% confidence intervals (95% CI) were calculated using the least square fit method. All calculations were performed using GraphPad Prism v 5.1 software (GraphPad Software, Inc., La Jolla, CA, USA). Freely available online GUSAR software (Institute of Biomedical Chemistry, Moscow, Russia) was used to predict the LC_50_ values for 48 h exposure of the new compounds [84].

### 4.5. Prediction of the Molecular Mechanism of Action and Toxicity

#### 4.5.1. PASS Prediction

A virtual screening was performed using the software PASS (Prediction of Activity Spectra for Substances), a product designed to evaluate the pharmacological potential of newly synthesized compounds. The structures were inputted in PASS as SMILES and the results were analyzed if the Pa values were above the corresponding Pi values.

#### 4.5.2. Structural Similarity Analysis

A similarity search was performed on the ChEMBL database for the compounds **3**, **4**, **5a**,**b**, and **6a**,**b** using a 50% threshold [81]. The resulting structures were extracted together with their assayed activities on bacteria [85]. The entries were filtered using DataWarrior v5.2.1 software [86] to remove duplicate structures.

## 5. Conclusions

In summary, new analogs from *N*-acyl-α-amino acids, 1,3-oxazol-5(4*H*)-ones, *N*-acyl-α-amino ketones, and 1,3-oxazoles classes bearing a 4-[(4-chlorophenyl)sulfonyl]phenyl moiety, were synthesized and physicochemically and biologically characterized. The antimicrobial activity evaluation revealed that the *N*-acyl-α-amino acid **3** and 1,3-oxazol-5(4*H*)-one **4** exhibited antimicrobial activity against Gram-positive bacterial strains and 1,3-oxazole **6a** against the *C. albicans* strain.

## 6. Patents

Patent application a201900668: Theodora-Venera Apostol, Stefania-Felicia Barbuceanu, Laura-Ileana Socea, Ioana Saramet, Constantin Draghici, Valeria Radulescu, Mariana Carmen Chifiriuc, Luminita Gabriela Marutescu, Octavian Tudorel Olaru, George Mihai Nitulescu, 4-Isopropyl-1,3-oxazol-5(4*H*)-one Derivatives Containing a Diaryl sulfonyl Substituent in Position 2 with Antimicrobial Action, published in RO-BOPI, 8/2020 from 28 August 2020.

## Figures and Tables

**Table 1:** Results of the qualitative assay of the antimicrobial activity of the tested compounds by using an adapted diffusion assay (the growth inhibition diameters were measured and expressed in mm).

Compound	Growth Inhibition Diameters (mm)					
	*Enterococcus faecium* E5	*Staphylococcus aureus* ATCC 6538	*Bacillus subtilis* ATCC 6683	*Pseudomonas aeruginosa* ATCC 27857	*Escherichia coli* ATCC 8739	*Candida albicans* 393
**3**	15	0	0	0	0	0
**4**	10	8	9	0	0	0
**5a**	0	0	0	0	0	0
**5b**	0	9	0	0	0	0
**6a**	0	0	0	0	0	8
**6b**	0	0	0	0	0	0
Ciprofloxacin	30	26	28	30	34	- ^1^
Flucozanole	-	-	-	-	-	30

^1^ -, not tested.

**Table 2:** Antibiotic susceptibility testing of the *E. faecium* E5 strain cultivated in the presence of DMSO and of sub-inhibitory concentrations of compounds **3** and **4**.

Microbial Culture	Diameter of Growth Inhibition Zone (mm)			
	Ampicillin	Penicillin	Linezolid	Vancomycin
Control	24	14	27	19
**3**	21	12	25	18
**4**	23	13	27	19
DMSO	22	0	23	18

**Table 3:** Antibiotic susceptibility testing of the *S. aureus* ATCC 6538 strain cultivated in the presence of DMSO and of sub-inhibitory concentrations of compound **4**.

Microbial Culture	Diameter of Growth Inhibition Zone (mm)							
	Azithromycin	Penicillin	Vancomycin	Linezolid	Trimethoprim-Sulfamethoxazole	Clindamycin	Rifampicin	Cefoxitin
Control	15	0	23	32	0	26	21	20
**4**	19	30	15	26	0	23	12	25
DMSO	13	0	18	28	0	20	19	15

**Table 4:** The MIC and MBEC values (µg/mL) obtained for the tested compounds using microdilution method.

Compound	MIC and MBEC (µg/mL)											
	*Enterococcus faecium* E5		*Staphylococcus aureus* ATCC 6538		*Bacillus subtilis* ATCC 6683		*Pseudomonas aeruginosa* ATCC 27857		*Escherichia coli* ATCC 8739		*Candida albicans* 393	
	MIC	MBEC	MIC	MBEC	MIC	MBEC	MIC	MBEC	MIC	MBEC	MIC	MBEC
**3**	500	125	>500	>500	>500	>500	>500	>500	>500	>500	>500	>500
**4**	500	125	125	125	125	500	>500	>500	>500	>500	>500	>500
**5a**	>500	>500	>500	>500	>500	>500	>500	>500	>500	>500	>500	>500
**5b**	>500	>500	>500	500	>500	>500	>500	>500	>500	>500	>500	>500
**6a**	>500	>500	>500	>500	>500	>500	>500	>500	>500	>500	>500	125
**6b**	>500	>500	>500	>500	>500	>500	>500	>500	>500	>500	>500	>500
Ciprofloxacin	0.62	0.62	0.15	0.15	<0.03	<0.03	0.15	0.15	0.012	0.012	- ^1^	-
Fluconazole	-	-	-	-	-	-	-	-	-	-	<0.12	<0.12

^1^ -, not tested.

**Table 5:** *Daphnia magna* bioassay: 24 h and 48 h values, 95% confidence intervals for newly synthesized compounds and their starting materials.

Tested Compound	Predicted LC_50 (48 h)_ ^1^ (µg/mL)	Determined LC_50 (24 h)_ (µg/mL)	95% CI ^2^ of LC_50 (24 h)_ (µg/mL)	Determined LC_50 (48 h)_ (µg/mL)	95% CI of LC_50 (48 h)_ (µg/mL)
**3**	3.12	ND * ^3^	ND *	ND *	ND *
**4**	1.86	ND *	ND *	44.00	42.84–45.19
**5a**	0.17	ND *	ND *	27.98	21.05–37.18
**5b**	0.22	58.62	42.98–79.94	5.57	3.752–8.269
**6a**	0.15	ND *	ND *	43.29	37.83–49.54
**6b**	0.19	ND *	ND *	45.17	39.48–51.69
Valine (control 1)	1078.3	ND *	ND *	ND *	ND *
**1** (control 2)	40.68	21.92	ND **	1.79	1.14–2.83

^1^ LC_50_—50% lethal concentration; ^2^ 95% CI—95% confidence interval; ^3^ ND—not determined due to lethality values; * the compound induced lethality values between 0 and 40%; ** 95% CI is too wide to be determined.

**Table 6:** The probability of compounds **3**, **4**, **5a**,**b**, **6a**,**b**, **7a**,**b**, and **8a**,**b** to be active (Pa) as antimicrobials as predicted by PASS analysis.

Compound	Target		
	Antimycobacterial	Antituberculosis	Antiinfective
**3**	0.39	0.40	0.54
**4**	0.49	0.34	0.35
**5a**	0.41	0.41	0.34
**5b**	0.42	0.41	0.38
**6a**	0.28	0.23	0.27
**6b**	0.28	0.24	0.31
**7a**	0.27	0.31	0.22
**7b**	0.27	0.31	0.26
**8a**	- ^1^	-	-
**8b**	-	-	-

^1^ -, the compounds were inactive.

## Data Availability

The datasets used and/or analyzed during the current study are available from the corresponding author on reasonable request.

## Sample Availability

Not available.
